# Assessment of Testicular Corticosterone Biosynthesis in Adult Male Rats

**DOI:** 10.1371/journal.pone.0117795

**Published:** 2015-02-23

**Authors:** Naoyuki Maeda, Sachi Tahata, Takeshi Yagi, Emi Tanaka, Kanae Masu, Michiko Sato, Satoko Haeno, Takenori Onaga, Hiroshi Yokota

**Affiliations:** 1 Laboratory of Veterinary Biochemistry, School of Veterinary Medicine, Rakuno Gakuen University Ebetsu, Hokkaido, Japan; 2 Japan Meat Science & Technology Institute, Shibuya-ku, Tokyo, Japan; 3 Laboratory of Veterinary Nutrition, School of Veterinary Medicine, Rakuno Gakuen University Ebetsu, Hokkaido, Japan; University Hospital of Münster, GERMANY

## Abstract

Corticosterone is synthesized in the adrenal glands and is circulated throughout the body to perform regulatory functions in various tissues. The testis is known to synthesize and secrete testosterone and other androgens. We developed an accurate method to measure steroid content using liquid chromatography-mass spectrometry analysis. In the present study, significant levels of the precursor compounds of testosterone and corticosterone synthesis could be detected in rat testis using this method. After adrenalectomy, corticosterone remained in the blood and testicular tissue at approximately 1% of the amount present in the control testis. When the excised testicular tissue was washed and incubated with NADH, NADPH and progesterone, not only testosterone and its precursors but also 11-deoxycorticosterone and corticosterone were produced; the levels of 11-deoxycorticosterone and corticosterone increased with incubation time. The production rate of 11-deoxycorticosterone from progesterone was estimated to be approximately 1/20 that of 17-hydroxyprogesterone, and the corticosterone level was approximately 1/10 that of testosterone. These ratios coincided with those in the testicular tissue of the adrenalectomized rats, indicating that corticosterone was synthesized in the testis and not in the blood. A primary finding of this study was that corticosterone and testosterone were synthesized in a 1/10-20 ratio in the testis. It is concluded that corticosterone, which has various functions, such as the regulation of glycolysis and mediating spermatogenesis, is produced locally in the testis and that this the local production is convenient and functional to respond to local needs.

## Introduction

Glucocorticoids and mineralocorticoids have been thought to be synthesized and secreted exclusively by the adrenal glands. Recently, evidence that the Sertoli cell glucocorticoid receptor (GR) is required to maintain normal testicular functions, circulating gonadotropin levels, optimal Leydig cell maturation and steroidogenesis was provided by Hazra et al. [1]. These findings suggest new insight into the glucocorticoid-mediated impact on male reproduction. However, the presence of steroidogenic enzymes and high local corticoid levels even after adrenalectomy indicate that corticosteroids can be locally synthesized in various tissues, such as those within blood vessels, the heart, and the brain [2–3]. Assessments of plasma steroids have been performed using RIA or ELISA with specific antibodies. As it is difficult to assay the amount of steroids in tissues and organs containing low steroid levels, research regarding glucocorticoids (corticosterone in rats) has focused on measuring circulating levels in the plasma or serum; however, direct evidence for the local biosynthesis of corticosterone in extra-adrenal tissues has not been obtained. Evidence has demonstrated local de novo synthesis of corticosterone in other organs; this was assessed via the expression of mRNA encoding enzymes that mediate corticosterone biosynthesis and via enzyme immunoblotting analysis [3–4]. Local corticosterone biosynthesis in extra-adrenal tissues is interesting, but the physiological importance remains unknown. The secretion of corticosteroids from extra-adrenal tissues raises the possibility of a novel paracrine or autocrine role for these hormones; it is necessary to establish that the biosynthetic apparatus and the hormone precursors are present and active in these tissues. Recently, research into extra-adrenal corticosteroid production has focused on the cardiovascular and the central nervous systems. However, mRNA and the enzymatic activity of 11β-hydroxylase, which mediates corticosterone biosynthesis from 11-deoxycorticosterone, were detected in Leydig cells purified from rat testis [5]. The authors concluded that the 11β-hydroxylase enzyme may be involved in the regulation of glucocorticoid metabolism within the testis through the local biosynthesis of endogenous inhibitors of 11β-hydroxysteroid dehydrogenase type 1 (11β-HSD1); this enzyme catalyzes the oxidation of glucocorticoids to inactive 11-dehydro metabolites [5]. As various steroids are synthesized and present in reproductive organs, an accurate and highly sensitive assay is necessary to investigate corticosteroid biosynthesis in the testis. We recently developed an accurate and highly sensitive assay for steroid detection using liquid chromatography/time-of-flight/mass spectrometry (LC-TOF MS) and liquid chromatography-tandem mass spectrometry (LC MS/MS) analysis; we were able to measure the levels of steroid hormones and precursors contained in several non-adrenal organs. These tissue steroid determination methods have a high sensitivity that is approximately 1,500-fold that of RIA [6]. We recently observed significant levels of 11-deoxycorticosterone in the testis [6], which suggests the possibility of the biosynthesis of corticosterone by the 11β-hydroxylase detected in the testis [5].

We aimed to confirm the testicular biosynthesis of corticosterone by detecting the precursors and the enzymatic activities mediating its biosynthesis and to assess the amount of locally synthesized corticosterone.

## Materials and Methods

### Chemicals and reagents

The following were purchased from Sigma-Aldrich (St. Louis, MO, USA): the steroid hormone corticosterone and its precursors including pregnenolone, progesterone, 11-deoxycorticosterone, dehydrocorticosterone, testosterone and its precursor, 17-hydroxyprogesterone, androstenedione and dihydrotestosterone; and stable isotopes, including testosterone-d3. The stable isotope corticosterone-d8 was purchased from Otsuka Pharmaceutical (Tokyo, Japan). LC-MS-grade acetonitrile and formic acid were purchased from Supelco (Bellefonte, PA, USA).

### Ethics Statement

This study was carried out in strict accordance with the recommendations in the Guide for the Care and Use of Laboratory Animals of the National Institutes of Health. The protocol was approved by the Committee on the Ethics of Animal Experiments of the Rakuno Gakuen University (Permit Number: VH23A14). All surgeries were performed under sodium pentobarbital anesthesia, and all efforts were made to minimize animal suffering.

### Preparation of rat organs

Ten male Sprague-Dawley (SD) rats (weight, 280 ± 20 g; age, 8–10 weeks) were fed, housed, and allowed to adapt to their environments for one week prior to the experiments. Blood and organ samples were collected from the animals via exsanguination under sodium pentobarbital anesthesia. After dissection, the excised testes were weighed post-mortem, immediately cut in half and thoroughly washed several times with phosphate-buffered saline to remove residual blood. Then, the washed testes were frozen and stored at -25°C until use.

### Adrenalectomy procedure

The first group of animals (n = 5) was bilaterally adrenalectomized under isoflurane anesthesia and allowed free access to 0.9% NaCl drinking water ad libitum for 2 weeks. The control group (n = 5) consisted of sham-operated animals drinking tap water. Removal of the adrenal glands was confirmed after the dissection of the rat and by the detection of aldosterone in the blood, as shown in Table 1.

**Table 1:** Concentrations of testosterone, corticosterone and aldosterone in the blood and testes of adrenalectomized adult male rats.

	Blood (pmoles/ml)			Testis (pmoles/g of tissue)		
	Control	Sham	AL	Control	Sham	AL
Testosterone	5.4±1.3	3.0±1.1	4.2±2.6	332.6±34.0	217.0±48.8	57.4±10.9**
Corticosterone	562.9±36.9	634.5±211.0	0.84±0.44**	73.6±32.2	1155.6±47.2	0.67±0.21**
Aldosterone	27.2±4.0	26.7±3.9	ND	ND	ND	ND

Adult male rats were adrenalectomized and bred for one week as described in the Materials and Methods section. Then, the testosterone, corticosterone and aldosterone concentrations in the blood and the testicular tissue were assayed, as described in the Materials and Methods section. ND; not detectable (Limit of detection; 5.0 fmoles/ml of blood or g of tissue). The data for each group represents the mean ± S.E.M of three to five rats.

**P<0.01 compared with the control rats.

### Determination of steroid levels expressed in the testis

Sample preparations for the MS analysis of the steroids were performed as previously described [6]. The LC-MS peaks corresponding to 11-deoxycorticosterone and corticosterone were identified using the SigmaFit algorithm, as shown in S1 and S2 Figs, and other steroids were identified as reported [6]. For the quantification of the steroid hormones, a TSQ Quantum Ultra triple-stage quadrupole mass spectrometer connected to an Ultimate 3000 (Thermo Fisher Scientific, San Jose, CA, USA) and an ESI source device was constructed (LC-MS/MS) as described [6]. The steroid hormones were used as the standards in the addition method [7]. The limits of detection of pregnenolone, progesterone, 17-hydroxyprogesterone, androstenedione, testosterone, 11-deoxycorticosterone and corticosterone were 14.2, 3.2, 2.8, 2.6, 0.6, 9.2 and 0.4 fmoles/g of tissue, respectively, as shown by Maeda [6].

### Assay for the enzyme reactions

To assess the enzymatic activities producing 11-deoxycorticosterone and corticosterone, half of the rat testis was washed with phosphate buffered saline at least three times, and the excised tissue was incubated with 30 μM progesterone in culture medium containing 4 mM MgCl_2_, 20 mM Tris-HCl buffer (pH 7.4), 100 μM NADPH and NADH at 37°C in a test tube. The enzyme activities were assayed as described by Wada et al. [8] with several modifications for the determination of the reaction product (11-deoxycorticosterone and corticosterone) using an MS analysis that we developed [6]. The reaction mixture (final volume of 1.0 ml) for the measurement of the enzymatic activity of 21-hydroxylase and 11β-hydroxylase contained 30 μM progesterone (as the substrate) and half of the testis. The reaction was initiated with the addition of 100 μM NADPH and NADH and incubated at 37°C for the desired time period; the mixture was then boiled to halt the enzyme reaction and vortexed thoroughly. Steroids (final concentration; 5.0 ng/ml) for the internal standards dissolved in 100 μl of acetonitrile containing 0.1% formic acid were added to the boiled mixture. The mixture was centrifuged at 10.160 x *g* for 10 min at 4°C and then 100 μl of hexane was added to the supernatant; the tube was shaken for 5 min at high speed. After centrifugation, the supernatants (10 μl) were used for MS analysis [6].

### Statistical analysis

Results are expressed as the mean ± S. E. M. of three to five independent experiments. Statistical analysis was carried out using the *F*-test and Student’s *t*-test.

## Results

We improved upon our previously reported simple method for preparing samples for LC-MS analysis to measure lipophilic compounds, such as steroids, in the blood and testicular tissue of rats [6]. This method has high sensitivity and accuracy (the detection value limit for corticosterone was only 0.4 f mol/ml). The detection and identification of corticosterone and 11-deoxycorticosterone in the testis were performed using the SigmaFit algorithm (Bruker software), as shown in S1 and S2 Figs, and the identified steroids were then quantified via LC-MS/MS analysis as described [6].

### Corticosterone in the adrenalectomized rat

**Fig 1:**
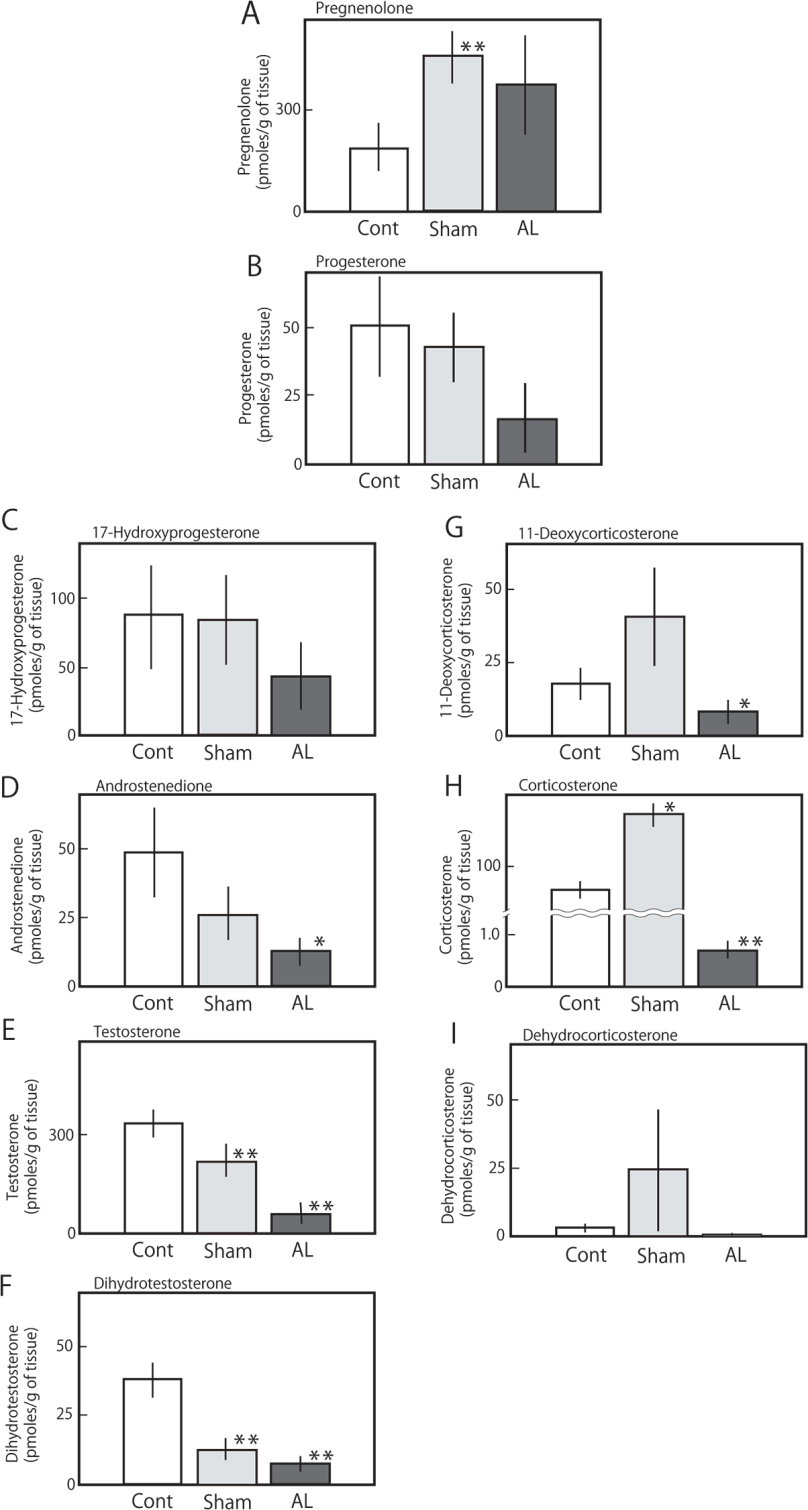
Steroid hormones and precursors in the testis of adrenalectomized rats. The adult male rats were adrenalectomized (AL) or sham-operated (Sham) as described in Materials and Methods. Two weeks after breeding, the levels of steroid hormones and their precursors were determined via LC-MS analysis, as described in Materials and Methods. The productions of pregnenolone (A), progesterone (B), 17-hydroxyprogesterone (C), androstenedione (D), testosterone (E), dihydrotestosterone (F), 11-deoxycorticosterone (G), corticosterone (H) and dehydrocorticosterone (I) are shown. The data for each group represents the mean ± S.E.M. of three to five rats. * P<0.05 and **P<0.01 compared with the control rats.

The blood and testicular testosterone and corticosterone levels in the adrenalectomized adult male rats were determined via LC-MS analysis (Table 1). A small amount of corticosterone remained in the blood and testis of the adrenalectomized rats. Aldosterone was not detected (the limit of detection was 5.0 fmoles/ml), and the adrenalectomy testis had approximately 1% of the amount of corticosterone observed in the controls. The blood and testicular corticosterone levels corresponded to 2% and 1% of the levels observed in the control rats, respectively. Interestingly, after adrenalectomy, the testosterone in the testis decreased to approximately 20% that of the controls (Table 1). The precursors of testicular steroidogenesis (11-deoxycorticosterone and corticosterone) could be detected in the testis, as shown in Fig. 1. All precursor compounds, with the exception of pregnenolone, decreased after the adrenalectomy (Fig. 1), suggesting that 3β-HSD, which produces progesterone from pregnenolone, was suppressed in the testes of the adrenalectomized rats. Corticosterone was drastically decreased in the testis (Fig. 1) as well as the blood (Table 1) after adrenalectomy; however, significant levels remained. The corticosterone level corresponded to 1/10 that of the testosterone in the same testis and 1/100–1/200 that of the corticosterone in the control testis, as shown in Table 1 and Fig. 1H. As corticosterone remained in the blood even after the adrenalectomy, the penetration of the blood corticosterone into the testicular tissue could not be ruled out. We performed several experiments after blood removal, as shown in the next section.

### Corticosterone production in testicular tissue after removing blood

**Fig 2:**
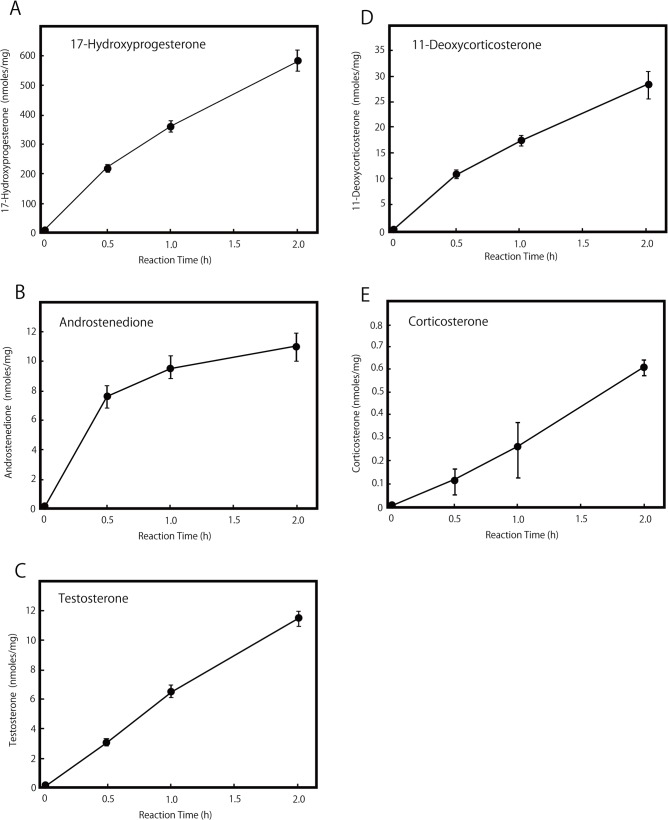
Enzyme activities of the synthesis steroids in rat testis. One half of the rat testis was washed with phosphate buffered saline at least three times, and the excised tissue was incubated with 30 μM progesterone in the culture medium containing 4 mM MgCl_2_, 20 mM Tris-HCl buffer (pH 7.4), 100 μM NADPH and NADH at 37°C in a test tube. The reaction products in the medium were determined using LC-MS analysis, as described in Materials and Methods. The contents of 17-hydroxyprogesterone (HPGS) (A), androstenedione (ADS) (B), testosterone (TS) (C), 11-deoxycorticosterone (DCC) (D), and corticosterone (CCS) (E) are shown.

**Fig 3:**
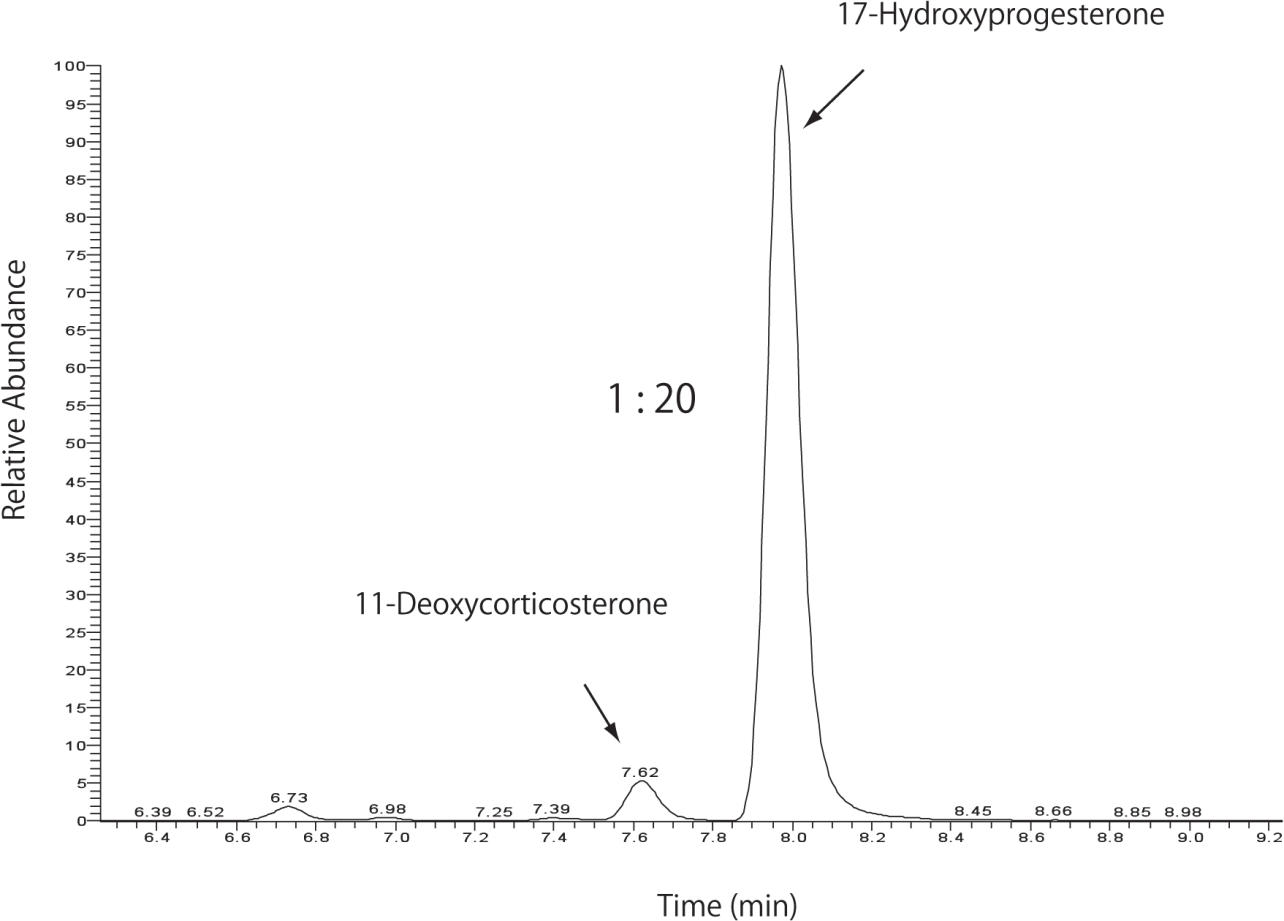
Production of 11-deoxycorticosterone and 17-hydroxyprogesterone in the enzyme assay using rat testis. After one hour of the enzymatic reaction described in Fig. 2, the production of 17-hydroxyprogesterone and 11-deoxycorticosterone was analyzed using LC-MS. Then, the two precursors with the same molecular weight in the same chromatogram were separated with LC; the ratio of the two products was 1:20.

**Fig 4:**
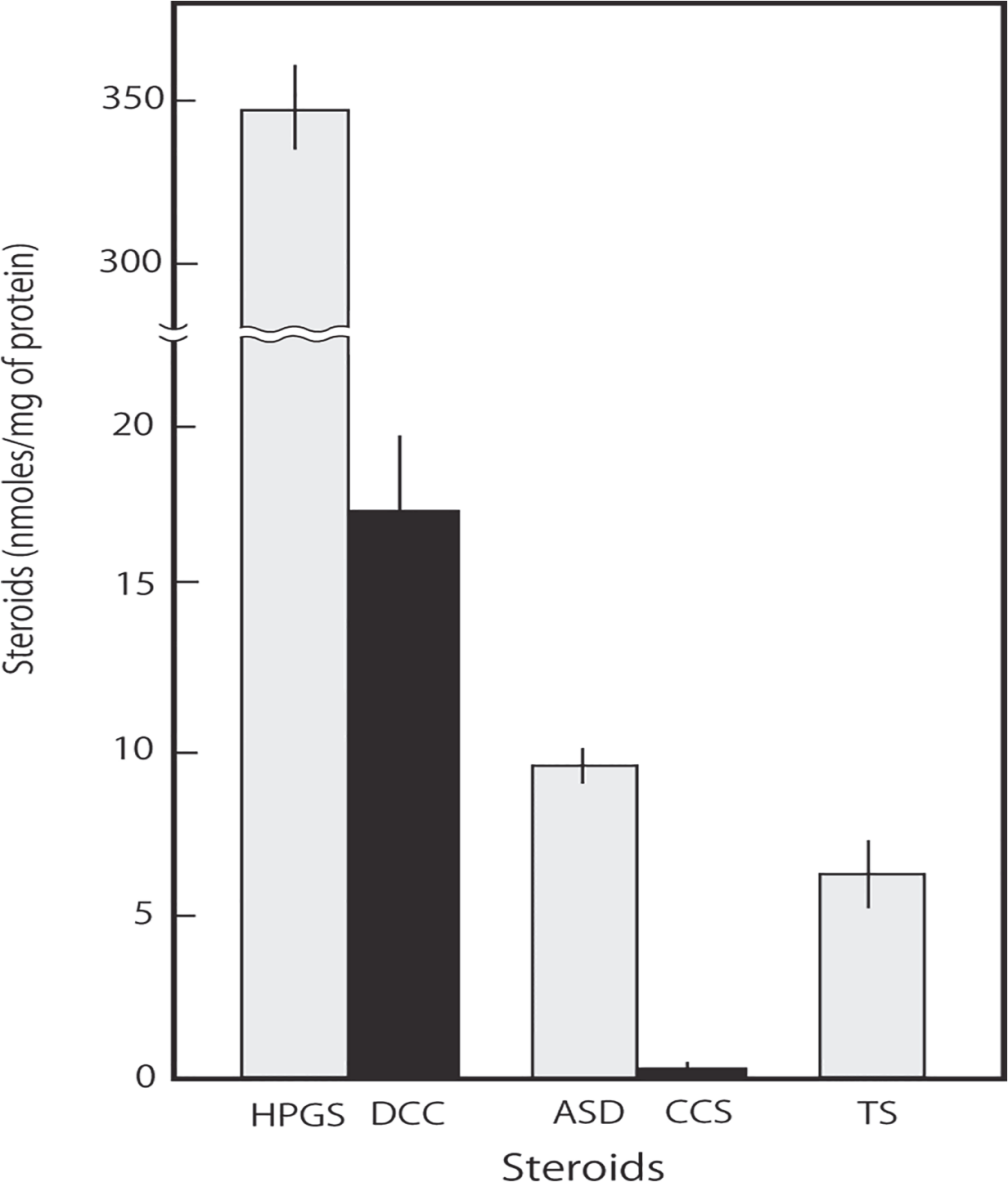
Amounts of the products in the enzyme assay using rat testis. The steroid production levels after the one-hour enzymatic reaction described in Fig. 2 are shown in the column figure. The data for each group represents the mean ± S.E.M. of three experiments.

In the present study, the majority of the blood that remained in the testis was removed by thoroughly washing the tissues cut in half several times with phosphate-buffered saline. However, it is impossible to omit the adrenal steroids incorporated into the testicular cells from the blood even by complete removal of blood. We next employed an in vitro assay to assess the enzymatic activities producing 11-deoxycorticosterone and corticosterone in the testis, as shown in Fig. 2. Excised rat testis was cultured with 30 μM progesterone, as described in Materials and Methods, and 11-deoxycorticosterone and corticosterone were detected in the culture medium and identified, as shown in S1 and S2 Figs. The concentrations of the products at each reaction time are shown in Fig. 2. 11-Deoxycorticosterone was produced from progesterone, indicating that the rat testis has steroid 21-hydroxylase activity (Fig. 2D). Corticosterone was also produced from progesterone, indicating that the testis has steroid 11β-hydroxylase activity, as previously shown [5] (Fig. 2E). The chromatogram of reaction products with the same molecular mass as 17-hydroxyprogesterone and 11-deoxycorticosterone is shown in Fig. 3. The product contents after one hour of semi-vivo enzyme reaction using testicular tissue are shown in Fig. 4. The conversion rates of progesterone to 17-hydroxyprogesterone and 11-deoxycorticosterone were approximately 20:1, as shown in Fig. 3. This ratio agreed with the ratio of the contents of 17-hydroxyprogesterone and 11-deoxycorticosterone (20:1) in the control testis, as shown in Fig. 1C and G; the same ratio of both reaction products is shown in Fig. 2A, D and Fig. 4. A small amount of corticosterone remained in the testis and was produced after the semi-vivo enzyme reaction using testicular tissue, as shown in Fig. 4. The ratio (1:20) of the produced corticosterone and testosterone levels was similar to the ratio (1:10) of the same steroids in the testis of the adrenalectomized rat, suggesting that these small amounts of corticosterone may correspond to the contents of the synthesized corticosterone in the testis.

## Discussion

### Adrenal corticosteroids and testicular steroidogenesis

Corticosterone is synthesized in the adrenal cortex, and steroid levels in blood are an important requirement and provide sufficient information for studying these hormones [7, 9]. In the present study, corticosterone and its precursors were detected in the adult rat testis. Testicular enzyme activity related to corticosterone synthesis was estimated using our developed LC-MS method, which was able to measure the levels of steroid hormones and their precursors contained in non-adrenal organs with approximately 1,500-fold greater sensitivity than RIA [6]. Our method can identify and determine the amounts of steroids synthesized locally after adrenalectomy. Western blot analysis with an antibody against P45011β and PCR sequencing data confirmed the expression of cytochrome P45011β in the Leydig cells of the rat testis, as reported [5]. We newly found steroid 21-hydroxylase in rat testis using in vivo and in vitro assays; this provides direct evidence supporting 11-deoxycorticosterone and corticosterone production in rat testes. These results indicate that corticosterone was synthesized in the rat testis, as shown in the brain and in other tissues [2, 10–11].

### Enzymes mediating corticosterone biosynthesis

Cytochrome P45011β and a gradual increase in the plasma 11-ketotestosterone level have been found in the testis during the sex differentiation of some protogynous fishes [12]. The authors suggested that testicular P45011β is involved in testicular differentiation by producing 11-ketotestosterone [12]. Rat Leydig cells express 11β-hydroxylase, which produces 11β-hydroxylated steroids. Testicular 11β-hydroxylase, an inhibitor of 11β-HSD1, may be used to control testosterone production. The physiological function of 11β-hydroxylase in the testis remains unknown; however, the local testicular synthesis of 11β-HSD1 inhibitors may provide another point of control in stress-induced reproductive dysfunction [9]. Steroid 21-hydroxylase mediated by P450C21 converts progesterone to 11-deoxycorticosterone and converts 17-hydroxyprogesterone to 11-deoxycortisol in the biosynthesis of aldosterone and cortisol. However, Mellon et al. showed that the protein and mRNA of P450C21 were not detected in the testis [13]. The extra-adrenal progesterone 21α-hydroxylation was catalyzed by two isoforms of the cytochrome P450 protein, CYP2C19 and CYP3A4, both with lower substrate affinities in patients having CYP2C19 mutations [14]. Testicular 21-hydroxylase activity was observed in the *in vitro* assay in our study (Figs. 2, 3 and 4), suggesting that 21-hydroxylase might be mediated in rat testis by a P450 isoform that was not P450C21, as described by Mellon et al. [13]. A small amount of 11-deoxycortisol (fmoles/mg of tissue levels) could be detected as a product from 11-deoxycorticosterone used in the enzymatic assay as a substrate (S3 Fig), but it could not be detected from progesterone used as a substrate in the enzymatic assay, as shown in Fig. 2. 11-Deoxycortisol and cortisol could also not be detected in the testicular tissue in vivo. These data suggest that the rat testis might have latent activities that produce 11-deoxycortisol and cortisol but that 17-hydroxyprogesterone was preferentially converted to androstenedione and testosterone (nmoles/mg of tissue levels).

### Advantage of the local biosynthesis of steroid hormones

The local synthesis of corticosteroids may have crucial functions in maintaining the homeostasis of the respective tissues. It is possible that the locally produced steroids act on the testicular cells via paracrine or autocrine systems with tissue-specific actions. Dexamethasone has been shown to suppress CRH expression, leading to a decrease in pituitary ACTH secretion [15]. It is interesting that ACTH stimulates the adrenal synthesis of corticosterone, suggesting that the local testicular production of corticosterone may be directly down-regulated by ACTH or adrenal corticosteroids. The effects of CRH and ACTH on the local testicular production of corticosterone are very interesting for investigating the regulation system and physiological functions. Corticosterone plays various roles in cells, including the regulation of glycolysis and glycogenesis. Adrenal corticosterone is regulated by ACTH via the HPA axis and is accepted only by cells with type I or type II receptors, whereas local production may be controlled based on the needs of each cell, such as those associated with energy production. This dual-directional process affects energy regulation, such as that associated with glycogen phosphorylase by serum epinephrine throughout the body and ATP concentrations within cells. The biosynthesis of testosterone in Leydig cells is strictly dependent on luteinizing hormone. However, testosterone biosynthesis can be directly inhibited by excessive glucocorticoid (corticosterone in rats), which could be caused pathologically by Cushing’s syndrome or psychologically by stress [7, 16]. The stress-induced increase in corticosterone secretion resulted in apoptosis in Leydig cells [17–18]. Glycolysis regulation is needed periodically for performing testicular functions, such as spermatogenesis, and the testicular production of corticosterone may play a role in local metabolic regulation. Hazra et al. demonstrated that Sertoli cell glucocorticoid receptor (GR) is required to maintain normal testicular functions, circulating gonadotropin levels, optimal Leydig cell maturation and steroidogenesis. These results mean that glucocorticoids play important roles in testicular functions, and it suggests that testicular local production of glucocorticoids is convenient and functional for responding to the local necessities in Sertoli cells and Leydig cells.

### Adrenal effects on testicular synthesis of corticosterone

Stress is a strong inducer of corticosterone excreted from the adrenal glands and suppresses testicular steroidogenesis, as shown by Whieledge and Cidlowski [19]. The data in Table 1 and Fig. 1 suggest that a certain stress was induced by the sham operation and that this stress could not be removed within one week after the operation. In Table 1, even after adrenalectomy, significant amounts of corticosterone and 11-deoxycorticosterone remained not only in the blood but also in the testis, as has been shown in the brain [20–21]. Blood corticosterone levels were approximately 10-fold higher than that in the testis, as shown in Table 1. In the adrenalectomized rats, the blood levels of not only corticosterone but also testosterone were drastically decreased. These results indicate that a significant change in the testicular level of the steroid was not dependent on the blood levels and suggests that the severe decrease in testicular steroids was dependent on the testicular conditions. Glucocorticoid deficiency due to Addison’s disease or adrenalectomy suppressed testicular testosterone production via the inhibition of various steroidogenic enzymes [19]. In Fig. 1, testicular testosterone, corticosterone and all of their precursors (with the exception of pregnenolone) were drastically decreased in the adrenalectomized adult male rats, indicating that testicular steroidogenesis was reduced *via* the decrease in 3β-HSD activity and that 11-deoxycorticosterone and corticosterone are synthesized from progesterone locally in the testis. These results further suggest that testicular corticosterone production is regulated by adrenal corticosteroids; this finding is similar to that of a previous report that found that testosterone production in rat Leydig cells was induced by aldosterone [22].

Finally, corticosterone corresponding to 1/10–1/20 of the amount of testosterone is locally synthesized in the rat testis.

## Supporting Information

S1 Fig Identification and determination of corticosterone using the SigmaFit algorithm and LC-TOF MS analysis. (EPS) Extracted ion chromatogram of standard corticosterone monoisotopic mass with a proton width ± 0.005 Da (A) and in testis (B). The mass spectrum of the preparations from the testis via LC-TOF MS analysis developed in this study (C) and the theoretical levels of corticosterone containing an isotope (D). A small δ-value (sigma value) was observed at 0.0234, and the mass error was only 1.9 mDa, indicating the presence of corticosterone in the sample (E).
(EPS)

S2 Fig Identification and determination of 11-deoxycorticosterone using the SigmaFit algorithm and LC-TOF MS analysis. (EPS) Extracted ion chromatogram of standard 11-deoxycorticosteorne monoisotopic mass with a proton width ± 0.005 Da (A) and in the testis (B). The mass spectrum of the preparations from the testis via LC-TOF MS analysis developed in this study (C) and the theoretical abundances of 11-deoxycorticosterone containing an isotope (D). A δ-value (sigma value) between 0 and 1.0 shows higher identification. A small δ-value was observed at 0.0261, and the mass error was only 1.1 mDa, indicating the presence of 11-deoxycorticosterone in the sample (E).
(EPS)

S3 Fig Enzyme activity of 11-deoxycortisol synthesis in rat testis. (EPS) One half of the rat testis was washed with phosphate buffered saline three times, and the excised tissue was incubated with 30 μM 11-deoxycorticosterone in culture medium containing 4 mM MgCl_2_, 20 mM Tris-HCl buffer (pH 7.4), 100 μM NADPH and NADH at 37°C in a test tube. The reaction products in the medium were determined using LC-MS analysis as described in Materials and Methods. A small amount of 11-deoxycortisol (fmoles/ mg of tissue levels, LOD; 2.1 fmoles/ml) was produced and increased during the reaction time.
(EPS)
